# Comparison of validation and application on various cardiovascular disease mortality risk prediction models in Chinese rural population

**DOI:** 10.1038/srep43227

**Published:** 2017-03-24

**Authors:** Changqing Sun, Fei Xu, Xiaotian Liu, Mingwang Fang, Hao Zhou, Yixiao Lian, Chen Xie, Nan Sun, Chongjian Wang

**Affiliations:** 1 Department of Social Medicine and Health Management, College of Public Health, Zhengzhou University, Zhengzhou, Henan, PR China; 2 Department of Epidemiology and Biostatistics, College of Public Health, Zhengzhou University, Zhengzhou, Henan, PR China; 3 Department of Health Education, West China School of Public Health, Sichuan University, Chengdu, Sichuan, PR China; 4 Department of Management Information Systems, Terry College of Business, University of Georgia, Athens, Georgia, United State

## Abstract

This research aims to assess application of different cardiovascular disease (CVD) mortality risk prediction models in Chinese rural population. Data was collected from a 6-year follow-up survey in rural area of Henan Province, China. 10338 participants aged 40 to 65 years were included. Baseline study was conducted between 2007 and 2008, and followed up from 2013 to 2014. Seven models: general Framingham risk score (general-FRS), simplified-FRS, Systematic Coronary Risk Evaluation for high (SCORE-high), SCORE-low, Chinese ischemic CVD (CN-ICVD), Pooled Cohort Risk Equation for white (PCE-white) and for African-American (PCE-AA) were assessed and recalibrated. The model performance was evaluated by C-statistics and modified Nam-D’Agostino test. 168 CVD deaths occurred during follow-up. All seven models showed moderate C-statics ranging from 0.727 to 0.744. Following recalibration, general-FRS, simplified-FRS, CN-ICVD, PCE-white and PCE-AA had improved C-statistics of 0.776, 0.795, 0.793, 0.779, and 0.776 for men and 0.756, 0.753, 0.755, 0.758 and 0.760 for women, respectively. Calibrations χ^2^ of general-FRS, simplified-FRS, SCORE-high, CN-ICVD and PCE-AA model for men, and general-FRS, CN-ICVD and PCE-white model for women were statistically acceptable, indicating these models predicts CVD mortality risk more accurately than others and could be recommended in Chinese rural population.

Cardiovascular disease (CVD) is a leading cause of premature death and disability worldwide which responsible for more than 17 million deaths annually, with approximately 80% of the disease burden in low- and middle-income countries, such as China^1^. In order to decrease the mortality of CVD, efficient primary prevention strategies targeting at the individuals “at risk” are necessary^2^. CVD mortality risk prediction models, which utilize data of multiple risk factors, are ideal and cost-effective approach for making rational decisions regarding primary preventive strategy and clinic practice to identify and treat high-risk populations^3^.

There are various CVD risk score models that enable the quantification of CVD mortality risk in different countries and regions around the world^4^,^5^,^6^. However, few studies in the application of these models in China have been seen in report, let alone the application to Chinese rural population. As a developing country, China has more than 50% of the population living in rural areas where medical and health facilities are limited. Independent external validations of different CVD risk prediction models in Chinese rural population can provide more information on primary prevention of cardiovascular diseases.

The aim of this study is to compare and validate the performances of different CVD risk prediction models in Chinese rural population. Seven models will be assessed to examine whether existing models are adapted to settings of Chinese people.

## Results

### Baseline Characteristics

The demographic and clinical characteristics of the participants at baseline examination are presented in Table 1. Cardiovascular risk factors, such as total cholesterol (TC), high density lipoprotein cholesterol (HDL-c), fasting glucose, systolic blood pressure (SBP) and body mass index (BMI) were more prevalent in women than in men. However, smoking was prevalent in men and uncommon in women. The prevalence of hypertension and diabetes were higher in women than in men.

During a 6-year follow-up, 417 deaths from all-causes and 168 deaths due to CVD occurred in cohort with a follow-up duration of 60942 person-years. The 6-year cardiovascular and all-cause mortality rate was 1.6% and 4.0% respectively. There were 80 cardiovascular deaths in duration of 23406 person-years follow-up in men and 88 deaths in duration of 37536 person-years follow up in women. The average 6-year risk of CVD death was 1.71% for men and 1.17% for women.

### Cardiovascular risk stratification and mortality distribution

**Figure 1:**
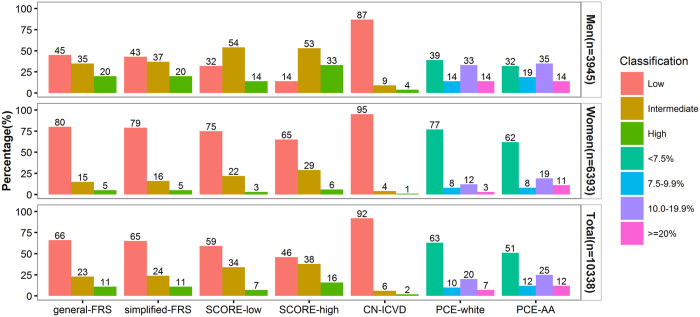
Comparison of cardiovascular risk categories for the FRS, SCORE, PCE and CN-ICVD prediction models (y-axis reflects percentage of individuals).

All seven original models were assessed, however, the ability of discrimination and calibration were poor (results were not shown). After FRS, CN-ICVD and PCE model recalibrated by mean values from Chinese present study and SCORE model recalibrated by diabetes status, the distributions of 10-year CVD death risk categories predicted by seven models (general-FRS, simplified-FRS, SCORE-low, SCORE-high, CN-ICVD, PCE-white and PCE-AA model) were present in Fig. 1.

As the PCE-white and PCE-AA model categorized the 10-year CVD death risk into four sets instead of three as the other five models did, their risk distribution was peculiar compared with others’. The FRS, SCORE, and CN-ICVD models indicated similar risk stratification trends in women. Despite the high prevalence of cardiovascular risk factors in the population, the SCORE-Low and CN-ICVD models divided more than 90% of the subjects into low-risk group, and in the SCORE-high and both FRS models the proportion of low risk was more than 60%. However, all risk models categorized quite different risk set in men.

### Comparison of CVD risk prediction models

The CVD mortality risk sets of PEC models were compared with that of FRS, SCORE and CN-ICVD models (see Supplementary Table S1, Supplementary Table S2 and Supplementary Table S3). The PCE-white model performed better agreement than PCE-AA model. The CN-ICVD model had a better agreement in low risk set than high. Agreement for CVD mortality risk categorization and correlation of scores between FRS models and SCORE models was good for both genders, and slightly better for men. There was hardly any misclassification between the extremes of risk categories in these models (see Supplementary Table S4). There was poor correlation between all models with the CN-ICVD model.

### Model performance

**Figure 2:**
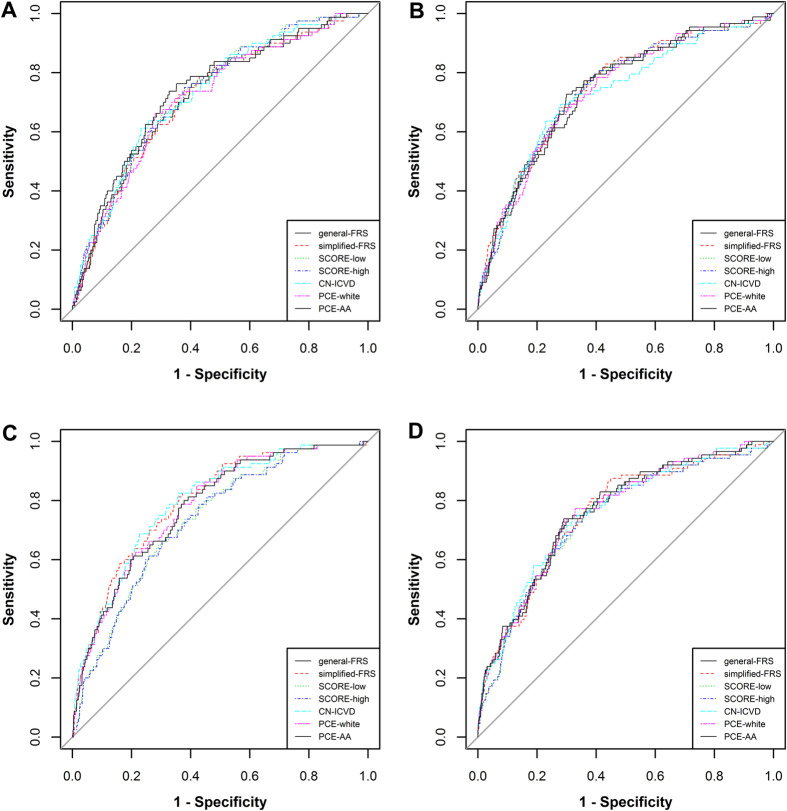
ROC curves of the FRS, SCORE, CN-ICVD and PCE models for prediction of cardiovascular mortality in men (**A**) and women (**B**), and ROC for seven recalibrated models in men (**C**) and women (**D**).

The receiver operating characteristic (ROC) curves of diffident CVD risk prediction models were shown in Fig. 2(A and B), with diacritical type of lines. Area under the ROC (AUC) was an indicator of the predictive veracity for the models. AUCs of seven CVD death prediction models showed moderately good discrimination for cardiovascular mortality (see Table 2). In men, the AUCs arranged from 0.714 (95%CI, 0.700–0.728) for simple FRS model to 0.736 (95%CI, 0.722–0.750) for PCE-AA model. Simplified-FRS model had a lower AUC compared with PCE-AA model (*P* = 0.013) for men. In women, the AUCs arranged from 0.732 (95%CI, 0.721–0.744) for SCORE-high model to 0.747 (95%CI, 0.736–0.758) for PCE-AA model.

SCORE-high model for men and PCE-white model for women showed good agreement between the predicted and Kaplan-Meier adjusted observed mortality events (see Supplementary Table S5, Supplementary Table S6 and Supplementary Figure S1). In men SCORE-low and PCE-AA model underestimated the CVD risk; however, PCE-white model overestimated the risk. Rather, in women the SCORE-low, PCE-AA model and SCORE-high model underestimated the CVD risk. Statistically, calibration of the SCORE-high model was acceptable for men with a modified Nam-D’Agostino test (χ^2^ = 5.109, *P* = 0.276), and so was PCE-white model for women with a modified Nam-D’Agostino test (χ^2^ = 2.310, *P* = 0.679).

### Coefficients Recalibration

Recalibration was conducted in five models (FRS, CN-ICVD and PCE models) by using the coefficients and mean values of risk factors recalibrated from the current population (Table 3 and Table 4). The ability of discrimination and calibration were evaluated in these models for 5-year CVD mortality (Table 5). ROC curves of diffident recalibrated CVD risk prediction models were shown in Fig. 2(C and D), Following recalibration general-FRS, simplified-FRS, CN-ICVD, PCE-white and PCE-AA had improved C-statistics of 0.776 (95% CI, 0.763–0.789), 0.795 (95% CI, 0.782–0.807), 0.793 (95% CI, 0.780–0.805), 0.779 (95% CI, 0.766–0.792), and 0.776 (95% CI, 0.763–0.789) for men and 0.756 (95% CI, 0.746–0.767), 0.753 (95% CI, 0.742–0.763), 0.755 (95% CI, 0.745–0.766), 0.758 (95% CI, 0.747–0.768) and 0.760 (95% CI, 0.750–0.771) for women, respectively. However, the change in SCORE models were not observed. Calibrations χ^2^ of general-FRS, simplified-FRS, SCORE-high, CN-ICVD and PCE-AA model for men, and general-FRS, CN-ICVD and PCE-white model for women was acceptable, respectively (see Supplementary Table S7, Supplementary Table S8 and Supplementary Figure S2).

## Discussion

The present study evaluated the ability of seven cardiovascular mortality risk models to predict CVD mortality risk in Chinese rural population. Predicted 10-year mortality risk distribution demonstrated that the general-FRS, simplified-FRS, SCORE-low, SCORE-high, PCE-white and PCE-AA models, while CN-ICVD model can stratify CV risk in Chinese rural population. Recalibration improved the model performance. All the seven models performed well in discrimination. Following recalibration, the general-FRS, simplified-FRS, SCORE-high, CN-ICVD and PCE-AA model could be recommended for men, and general-FRS, CN-ICVD and PCE-white model for women to predict CVD mortality risk in Chinese rural population.

Risk prediction models are essential and cost-effective for prevention of CVDs, especially in limited resource settings such as rural regions of China. The CN-ICVD model incorrectly categorized most people into low CVD mortality risk group in present study. This would lead high-risk individuals to be unidentified, resulting in higher rates of under-treatment and more subsequent complications. The findings of this study confirms that before the application of these prediction models to clinical practice or guidelines, the performances have to be assessed in the population interest as not all the risk-prediction models can identify high-risk subjects. Recalibrate the coefficients of models by the targeted population could improve the performance of predictive accuracy.

General-FRS model showed a good discrimination in Australian population^7^, Spanish population^8^ and Tehran population^9^, in which the AUCs were higher than the findings in this study. However, in a Malaysia hospital-based study, the general-FRS had a c-statistic of 0.63^10^, and this maybe because the study subjects were actual patients from a clinic but not general population from community, whose overall CVD risk profile was already high even before CVD events occurring. Simplified-FRS model also well discriminated in different studies^11^,^12^, which was similar to the findings of this study. General-FRS and simplified-FRS were both assessed and no difference was observed between them, indicating that the simplified-FRS model without laboratory parameters can be used more widely in low-income regions. SCORE models performed similarly in European countries from which they developed^13^,^14^. However, the SCORE-high model performed poorly in Norway, a high cardiovascular risk country^15^, suggesting that the validation of a recommended model before application is essential for every country. The calibration statistics reported previously indicated the SCORE-high model was well-calibrated in Asian males^12^, but not in females, consistent with our study. The CVD death rate is quite low in this population while the SCORE-high model had a well performance in men. This may be related to the observation was 6 years instead of 10 years in original study. In addition, the CVD mortality would be high in 10-years follow-up, which needs furthermore researches to validate. The SCORE models performing poorly on calibration in women may be as a result of the underestimation of women’s cardiovascular mortality risk for Chinese women have shown high cardiovascular causes of mortality^16^. The performances of PCE models were evaluated in the Korean Heart Study population, showing an AUC of 0.727 (PCE-white) and 0.725 (PCE-AA) for men, the corresponding AUC for women were 0.738 and 0.739^17^. While the study in Malaysian population showed a moderate discrimination with AUC of 0.63 and a good calibration with χ^2^ = 12.6 (*P* = 0.12)^18^. In this study, both the PCE models had a good discrimination and PCE-white model calibrated well with calibration χ^2^ for women.

Despite the mainly Caucasian ethnicity in development of the cardiovascular risk prediction models (FRS, SCORE and PCE models) assessed in this study, following recalibration they were able to discriminate cardiovascular risk in Chinese rural population. This is most likely because these prediction models were developed from contemporary real population cohorts and contemporarily used in other countries with good discrimination. Finally, the recalibrated general-FRS, simplified-FRS, SCORE-high, CN-ICVD and PCE-AA model could be recommended for men, and general-FRS, CN-ICVD and PCE-white model for women to predict CVD mortality risk in Chinese rural population.

### Perspectives

The early identification of high-risk individuals is a crucial strategy in primary prevention of cardiovascular diseases (CVD). Effective implementation of a strategy to identify these individuals in a clinical setting is reliant on the availability of appropriate CVD risk prediction models and guideline recommendations. Several well-known models for CVD mortality risk prediction have been developed and utilized in the USA and Europe, but might not be suitable for use in other regions or countries.

In this study, seven CVD mortality risk prediction models were assessed and recalibrated in rural population. General-FRS, simplified-FRS, SCORE-high, CN-ICVD and PCE-AA model for men, and general-FRS, CN-ICVD and PCE-white model for women predicts CVD mortality risk in Chinese rural population more accurately than others, those could be recommended in clinical practice or guidelines. It might be helpful to rural health care practitioners and people to predict the risk of CVD as well as improving preventive awareness of CVD. The following study will focus on developing a CVD mortality risk prediction model for Chinese rural population.

### Strengths and Limitations

This study collected data from a relatively large scale population-based prospective cohort in Chinese rural regions. Although it is the first time to assess seven CVD deaths risk prediction in Chinese rural population, some limitations need to be noticed. First, the mortality information was obtained from the national death registration record. Since the record system was not perfect, there might be some missing death events, which was confirmed by physician of the local clinic. Second, data were collected on actual observed 6-year CVD mortality events, instead of 10-year predicted in seven original studies. Calibrations of the SCORE and PCE models were calculated by adjusting coefficient to predict 5-year CVD mortality risk, and we recalibrated the coefficients and baseline survival rate for 5 years Third, the study was conducted in single area of Henan Province, therefore, the results need to be validated on a larger population, probably in a multicenter study. Although this study has several limitations, the findings are relatively actual and reliable to reflect the real condition.

**Novelty and Significance.** 1) What Is New? Different CVD mortality risk prediction models have been developed and widely validated in western countries. However, research on validation of these existing risk models in Chinese population is limited, especially in rural regions. Our study assessed and recalibrated seven CVD morality risk prediction models in Chinese rural population. The main findings suggested that recalibration of model could improve the ability of discrimination and calibration. Following recalibration general-FRS, simplified-FRS, SCORE-high, CN-ICVD and PCE-AA model for men, and general-FRS, CN-ICVD and PCE-white model for women predicts CVD mortality risk more accurately than others, and those could be recommended in Chinese rural prediction (could be used to clinical practice or guidelines).2) What Is Relevant? Findings of this study might be helpful to rural health care practitioners and people to predict the risk of CVD as well as improving preventive awareness of CVD. Moreover, the availability of appropriate CVD mortality risk model is essential to reduce CVD deaths via identifying high risk individuals, which can urge them to change life-style and get treatment if necessary. And the early identification of high-risk individuals is effective on health education.

## Conclusion

The study highlighted that it is crucial to assess and recalibrate cardiovascular mortality risk prediction models before their application to clinical practice or guidelines, as not all the risk-prediction models can distinguish between high and low-risk objects. Following recalibration, general-FRS, simplified-FRS, SCORE-high, CN-ICVD and PCE-AA model for men, and general-FRS, CN-ICVD and PCE-white model for women can be used to identify high cardiovascular risk in the Chinese rural population. The application of existing prediction models for CVD mortality should be cautious and it is essential to assess and recalibrate the original models in targeted population.

## Methods

### Study population and samples

This survey is a 6-year population-based prospective cohort study on the rural areas in Henan Province, China. Details of survey methods have been described and reported previously^19^. Briefly, the baseline survey was conducted from July to August of 2007 and that of 2008, and the data were collected by questionnaires, medical examinations and fasting blood samples. Subjects were permanent residents with no major disability or severe infectious diseases. Follow-up survey was completed in the same way from July to August of 2013 and July to October of 2014. There were 20194 participants aged 18 to 78 years in original cohort. Participants with a baseline age outside the age range of interest were excluded from this study (4401 persons <40 years; 3030 persons >65 years). There were 1531 participants not coming for follow-up in the 6-year duration. Out of 11232 participants, 864 participants with cancer, chronic kidney disease or prior history of CVD at baseline were excluded. Another 30 participants were also excluded as there were missing data for calculation of risk models. Eventually a total of 10338 participants were eligible for analysis. This study was approved by the Medical Ethics Committee of Zhengzhou University. The methods were carried out *in accordance with* the relevant guidelines and regulations. All participants signed an informed-consent form.

### Data Collection and Laboratory Measurements

Data were collected by specially trained physicians and public health workers who used standardized methods with stringent quality control. The information regarding demographic characters, family and individual disease history, dietary and lifestyle were obtained by a standardized questionnaire. Anthropometric data were also taken: height, weight, waist circumference and hip circumference measured twice, systolic blood pressure (SBP) and diastolic blood pressure (DBP) recorded utilizing HEM-770A sphygmomanometer in the sitting position for three times according to the American Heart Association’s standardized protocol^20^. Eight to ten hours of fasting blood specimens were collected in EDTA-K_2_ tubes for measurement of lipid profile and plasma glucose, respectively. Blood specimens were centrifuged at 4 °C and 3,000 rpm for 10 minutes, and the plasma was transferred and stored at −20 °C for biochemical analyses. Hypertension was defined as SBP ≥140 mm Hg and/or DBP ≥90 mm Hg, and/or diagnosed as hypertension by a physician and currently receiving anti-hypertension treatment according to 20l0 Chinese guidelines for the management of hypertension^21^. Diabetes status was defined as having a fasting plasma glucose (FPG) ≥7.0 mmol/L, and/or diagnosed as diabetes by a physician^22^. Type 1 diabetes mellitus, gestational diabetes and other special type diabetes were excluded.

### Cardiovascular Disease outcomes

The cardiovascular mortality was the outcome of interest in this study. Mortality information was obtained and confirmed from the national death registration record and physician of the local clinic. Missing morality causes were classified as unexplained deaths. Death caused by CVD is coded by specialized physician in the International Classification of Diseases (ICD)-10 codes I10–I15, I20–25, I60–I69, I70 and I71 or in ICD-9 codes 401–414, 426–443, 798.1 (instantaneous death) and 798.2 (death within 24 h of symptom onset) with the exception of 426.7, 429.0, 430.0, 432.1, 437.3, 437.4, and 437.5, which were used in the SCORE models^5^.

### Cardiovascular risk prediction models

Seven CVD mortality risk models were selected and validated in this survey: the Systematic Coronary Risk Evaluation (SCORE) models (include SCORE-high and SCORE-low model)^5^, the Framingham Risk Score (FRS) models (include general-FRS and simplified-FRS model)^4^, the American College of Cardiology (ACC) and American Heart Association (AHA) new pooled cohort risk equation (PCE) models (include PCE-white and PCE-AA prediction model)^6^,^23^, and a Chinese ischemic cardiovascular diseases risk model (CN-ICVD model)^24^ were included as they have similar risk factors and endpoints. All seven models predict the risk of 10-year CVD death. Furthermore, the risk factors used to calculate the CVD mortality risk for these seven models were collected in this study. Both SCORE models were included in this study as lack of information about whether the low risk or the high risk model performed better in China rural. The same with SCORE model, both PCE models were selected.

Diabetes status is not considered in the original SCORE models, whereas the risks predicted with diabetes were recommended to be multiplied two-fold for male and four-fold for female in SCORE models^5^. According to the current estimated effect of diabetes on CVD risk^25^, the predicted CVD death risk for individuals with diabetes was calculated multiplying by three in male, and by five in female in this study. Cardiovascular death risk and classified risk category of each subject for all seven models were calculated. For FRS, SCORE, and CN-ICVD models, cardiovascular risk was stratified into three categories: low, intermediate, and high. However, for both the PCE models, CVD risk was stratified into four categories^6^: <7.5%, 7.5–9.9%, 10.0–19.9% and ≥20%. Low CV risk was defined as ten-year risk of <10%, <1% and <5% for the FRS, SCORE and CN-ICVD models, respectively^4^,^5^,^24^. High risk was defined as ≥20% for both the FRS models^4^, ≥10% for CN-ICVD model^24^ and ≥5% for both the SCORE models^5^. All the other values were stratified into intermediate risk group. The Spearman’s correlation coefficients were also calculated to assess the correlation between the rankings of each participant’s absolute CV mortality risk. Lower numbers of population misclassification in extremes of risk categories were used to determine the agreement between different models.

### Statistical Analysis

The whole process of statistical analysis was performed with the R software (version 3.2.3, https://www.R-project.org). Continuous variables were described by mean ± standard deviation (if were normal distribution) or median (inter-quartile range) (if were not normal distribution), while categorical data were reported as count and percentages. The six-year predicted CVD mortality risk of each subject was calculated by the seven risk models, and then compared with the actual observed CVD deaths. Validity and the predictive accuracy of the CVD risk models were assessed based on their discrimination and calibration. A 2-tailed *P* value < 0.05 was considered significant.

The C-statistics was calculated to evaluate the discriminative power of risk models. C-statistics was also known as the area under the receiver operating characteristic (ROC) curve (AUC), and the ROC curve was plotted. Calibration was assessed statistically by modified Nam-D’Agostino test^26^,^27^ to determine if the observed cardiovascular deaths differed significantly from the expected^28^. The Kaplan-Meier analysis was used to obtain the number of observed CVD death event^1^, which was then compared with the predicted events in each group in calibration charts. Ideally a well- calibrated model performs well in a variety of divisions into groups. We started with 10 deciles, but collapsed small deciles with their closest neighbors, until all groups contained a predefined minimum number of events (at least 2 per group) according to the prior study by Demler and her colleagues^27^. Finally, all the seven models were evaluated by collapsing into five groups for women. All models were evaluated by collapsing into five groups except CN-ICVD model which was accessed by four groups. Calibration was also determined graphically by plotting the observed and expected mortality events, grouped according to five or four groups of predicted possibility, respectively.

### CVD models Recalibration

All seven original models were accessed while the ability of discrimination and calibration were poor (results were not shown). In the adjusted FRS, CN-ICVD and PCE model, the coefficients were taken from the original models, but mean values from Chinese current study were used for the risk factors and the mean incidence rates. As the mean variable of risk factor were not included in original SCORE models, thus the SCORE models were adjusted by diabetes status (see ***Cardiovascular risk prediction models***). Discrimination was calculated for all seven models and calibration was tested for four models: SCORE-high, SCORE-low, PCE-white and PCE-AA model. Only for model calibration, the 5-year risks of fatal CVD were estimated by modifying the S_0_(10) into S_0_(5) based on previous study^29^ in PCE models and adjusting all relevant regression equations by Conroy *et al*.^5^ in SCORE models.

In theory, a more appropriate integrated event risk prediction model could be produced by adopting coefficients to correct for different background incidence rates in different cultures^30^,^31^. Thus, recalibration was conducted based on adjustment of coefficients and mean value for each risk factor in this study. The coefficients and mean values were recalibrated from the Chinese current population if the original model was expressed in form of formula (1).


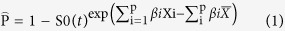


Finally, FRS, CN-ICVD and PCE models were recalibrated and assessed in this approach. The SCORE-high, SCORE-low were modified time parameter of the equations based on Conroy *et al*.^5^ and the risk factor of diabetes status.

## Additional Information

**How to cite this article**: Sun, C. *et al*. Comparison of validation and application on various cardiovascular disease mortality risk prediction models in Chinese rural population. *Sci. Rep.* **7**, 43227; doi: 10.1038/srep43227 (2017).

**Publisher's note:** Springer Nature remains neutral with regard to jurisdictional claims in published maps and institutional affiliations.

## Supplementary Material

Supplementary Information

## Figures and Tables

**Table 1:** Characteristics of Study Participants.

Variables	Overall	Men	Women	*P*
N, %	10338	3945 (38.16)	6393 (61.84)	<0.001
	**Mean ± SD**	**Mean ± SD**	**Mean ± SD**	
Age, years	52.01 ± 7.21	52.86 ± 7.23	51.49 ± 7.15	<0.001
Total cholesterol, mmol/L	4.58 ± 0.93	4.42 ± 0.87	4.67 ± 0.96	<0.001
High density lipoprotein, mmol/L	1.17 ± 0.26	1.11 ± 0.25	1.20 ± 0.26	<0.001
Fasting glucose, mmol/L	5.73 ± 1.59	5.60 ± 1.39	5.80 ± 1.71	<0.001
Systolic blood pressure, mm Hg	126.43 ± 19.90	124.69 ± 17.78	127.51 ± 21.07	<0.001
Body mass index, kg/m^2^	24.64 ± 3.54	23.79 ± 3.22	25.17 ± 3.62	<0.001
Waist circumference, cm	83.44 ± 10.09	83.62 ± 9.95	83.33 ± 10.18	0.1516
	**N, %**	**N, %**	**N, %**	
Age groups (years)				<0.001
40–49	3947 (38.18)	1352 (34.27)	2595 (40.59)	
50–59	4451 (43.05)	1724 (43.70)	2727 (42.66)	
60–65	1940 (18.77)	869 (22.03)	1071 (16.75)	
Education				<0.001
Illiteracy	1399 (13.53)	139 (3.52)	1260 (19.71)	
Primary school	3591 (34.74)	1105 (28.01)	2486 (38.89)	
Junior high school	4291 (41.51)	2043 (51.79)	2248 (35.16)	
Senior high school or above	1057 (10.22)	658 (16.68)	399 (6.24)	
Income^*^				<0.001
<CNY^#^1000	9696 (93.93)	3659 (92.84)	6037 (94.61)	
CNY1000-2999	491 (4.76)	225 (5.71)	266 (4.17)	
≥CNY3000	135 (1.31)	57 (1.45)	78 (1.22)	
Current smoker	2894 (27.99)	2876 (72.90)	18 (0.28)	<0.001
Drinking	1129 (10.92)	1097 (27.81)	32 (0.50)	<0.001
Diabetes	960 (9.29)	313 (7.93)	647 (10.12)	<0.001
Hypertension	3296 (31.88)	1016 (25.75)	2280 (35.66)	<0.001
Hypertension Treatment	2030 (61.59)	578 (56.89)	1452 (63.68)	<0.001

Data are % for categorical variables and mean (sd) for continuous variables. *Average monthly income, ^#^CNY: Chinese Yuan.

**Table 2:** Sensitivity, specificity and discriminative ability for the FRS, SCORE, CN-ICVD and PCE models for 5-year cardiovascular mortality.

Models	Cut-off^*^	Sensitivity (95%CI)	Specificity (95%CI)	Cut-off^#^	Sensitivity (95%CI)	Specificity (95%CI)	AUC (95%CI)
general-FRS							
Overall	20%	35.12 (27.9, 42.8)	89.81 (89.2, 90.4)	11.26%	64.29 (56.5, 71.5)	71.70 (70.8, 72.6)	0.732 (0.723, 0.740)
Men	20%	50.00 (38.6, 61.4)	81.22 (79.9, 82.4)	12.93%	76.25 (65.4, 85.0)	59.74 (58.2, 61.3)	0.719 (0.705, 0.733)
Women	20%	21.59 (13.5, 31.6)	95.08 (94.5, 95.6)	6.46%	76.14 (65.9, 84.6)	64.28 (63.1, 65.5)	0.740 (0.730, 0.751)
simplified-FRS							
Overall	20%	35.71 (28.5, 43.5)	89.35 (88.7, 89.9)	12.65%	61.90 (54.1, 69.3)	74.95 (74.1, 75.8)	0.734 (0.726, 0.743)
Men	20%	50.00 (38.6, 61.4)	80.36 (79.1, 81.6)	14.27%	72.50 (61.4, 81.9)	64.27 (62.7, 65.8)	0.714 (0.700, 0.728)
Women	20%	22.73 (14.5, 32.9)	94.88 (94.3, 95.4)	8.22%	68.18 (57.4, 77.7)	72.29 (71.2, 73.4)	0.747 (0.736, 0.758)
SCORE-low							
Overall	5%	25.60 (19.2. 32.9)	92.74 (92.2, 93.2)	1.32%	70.24 (62.7, 77.0)	66.12 (65.2, 67.0)	0.734 (0.725, 0.743)
Men	5%	37.50 (26.9, 49.0)	86.05 (84.9, 87.1)	2.3%	73.75 (62.7, 83.0)	62.43 (60.9, 64.0)	0.732 (0.718, 0.746)
Women	5%	14.77 (8.1, 23.9)	96.84 (96.4, 97.3)	0.56%	78.41 (68.4, 86.5)	61.92 (60.7, 63.1)	0.742 (0.731, 0.753)
SCORE-high							
Overall	5%	41.67 (34.1, 49.5)	84.11 (83.4, 84.8)	1.35%	80.95 (74.2, 86.6)	53.97 (53.0, 54.9)	0.727 (0.719, 0.736)
Men	5%	67.50 (56.1, 77.6)	67.53 (66.0, 69.0)	4.24%	75.00 (61.4, 84.0)	62.10 (60.5, 63.6)	0.730 (0.715, 0.743)
Women	5%	18.18 (10.8, 27.8)	94.27 (93.7, 94.8)	0.82%	79.55 (69.6, 87.4)	60.89 (59.7, 62.1)	0.741 (0.731, 0.752)
CN-ICVD							
Overall	10%	10.71 (6.5, 16.4)	98.10 (97.8, 98.4)	1.41%	73.81 (66.5, 80.3)	66.11 (65.2, 67.0)	0.735 (0.726, 0.743)
Men	10%	15.00 (8.0, 24.7)	96.64 (96.0, 97.2)	2.81%	63.75 (52.2, 74.2)	74.31 (72.9, 75.7)	0.730 (0.716, 0.744)
Women	10%	6.82 (2.6, 14.3)	99.00 (98.7, 99.2)	1.13%	69.32 (58.6, 78.7)	71.99 (70.9, 73.1)	0.731 (0.720, 0.742)
PCE-white							
Overall	20%	24.40 (18.1, 31.6)	93.25 (92.7, 93.7)	8.59%	67.86 (60.2, 74.8)	67.91 (67.0, 68.8)	0.729 (0.720, 0.737)
Men	20%	36.25 (25.8, 47.8)	86.80 (85.7, 87.9)	14.18%	66.25 (54.8, 74.6)	71.90 (70.5, 73.3)	0.715 (0.701, 0.729)
Women	20%	13.64 (7.3, 22.6)	97.21 (96.8, 97.6)	6.23%	65.91 (55.0, 75.7)	72.43 (71.3, 73.5)	0.740 (0.729, 0.750)
PCE-AA							
Overall	20%	38.10 (30.7, 45.9)	88.19 (87.5, 88.8)	9.68%	77.98 (70.9, 84.0)	62.14 (61.2, 63.1)	0.744 (0.736, 0.753)
Men	20%	40.00 (29.2, 51.6)	86.52 (85.4, 87.6)	12.35%	76.25 (65.4, 85.0)	64.76 (63.2, 66.3)	0.736 (0.722, 0.750)
Women	20%	36.36 (26.4, 47.3)	89.21 (88.4, 90.0)	9.68%	72.73 (62.2, 81.7)	70.02 (68.9, 71.2)	0.745 (0.722, 0.750)

*The cut off recommended by each original model. ^#^The cut off recommended by present population.

**Table 3:** Coefficients of recalibration for FRS, CN-ICVD models.

Model	Variable	Coefficient	
		Men	Women
**general-FRS**	Ln of Age (y)	5.1215	4.4913
	Ln of Total Cholesterol (mg/dL)	−0.2727	−0.9884
	Ln HDL–C (mg/dL)	−0.4596	−0.4972
	Ln of SBP if not treated (mm Hg)	3.2282	3.4325
	Ln of SBP if treated (mm Hg)	3.3088	3.4539
	Smoking	−0.4882	−3.0644
	Diabetes	0.6518	0.7695
	**Mean (Coefficient × Value)**	**32.45731**	**27.33517**
	**Baseline Survival (S_5_)**	**0.9914698**	**0.9934118**
**simplified-FRS**	Ln of Age (y)	4.6867	4.0899
	Ln of BMI (kg/m^2^)	−2.2998	−0.6864
	Ln of SBP if not treated (mm Hg)	3.4576	3.4689
	Ln of SBP if treated (mm Hg)	3.5505	3.4942
	Smoking	−0.5276	−3.0307
	Diabetes	0.7303	0.7639
	**Mean (Coefficient × Value)**	**27.67513**	**30.74361**
	**Baseline Survival** (**S**_**5**_)	**0.9914576**	**0.993261**
**CN-ICVD**	Age, y	0.0844	0.0878
	SBP, mm Hg		
	<120	0.0186	−0.3130
	120–129	Referent	Referent
	130–139	0.7914	0.1822
	140–159	1.6302	0.4560
	160–179	1.9703	0.9871
	≥180	1.8387	1.8207
	BMI, kg/m^2^		
	<24	Referent	Referent
	≥24	−0.3101	−0.0624
	Total cholesterol, mmol/L		
	<3.62	Referent	Referent
	3.62–5.16	−0.0059	−0.6806
	≥5.17	−0.2680	−0.8578
	Current smoker, yes/no	−0.4775	−3.3481
	Diabetes, yes/no	0.7168	0.8233
	**Mean (Coefficient × Value)**	**4.400718**	**3.976495**
	**Baseline Survival** (**S**_**5**_)	**0.9918239**	**0.9931033**

Abbreviations: HDL-C, high-density lipoprotein cholesterol; BMI, Body Mass Index; SBP, systolic blood pressure; Ln, natural logarithm.

**Table 4:** Coefficients of recalibration for PCE models.

Gender	Variable	White	African American
		Coefficient	Coefficient
**Women**	Ln Age (y)	−22.3973	−42.6070
	Ln Age, Squared	1.7899	N/A
	Ln Total Cholesterol (mg/dL)	15.0642	−0.9533
	Ln Age × Ln Total Cholesterol	3.5038	N/A
	Ln HDL–C (mg/dL)	5.1555	0.5844
	Ln Age × Ln HDL–C	−1.4068	−0.2728
	Ln Treated SBP (mm Hg)	3.4666	−34.6531
	Ln Age × Ln Treated SBP	N/A	9.4585
	Ln Untreated SBP (mm Hg)	3.4459	−36.7922
	Ln Age × Ln Untreated SBP	N/A	9.9831
	Current Smoker (1 = Yes, 0 = No)	17.8261	−3.8740
	Ln Age × Current Smoker	−5.6228	N/A
	Diabetes (1 = Yes, 0 = No)	0.7707	0.7789
	**Mean (Coefficient × Value)**	−**51.67933**	**−162.2403**
	**Baseline Survival (S_5_)**	**0.9934143**	**0.9931984**
**Men**	Ln Age (y)	−7.6938	5.1215
	Ln Total Cholesterol (mg/dL)	−20.8736	−0.2727
	Ln Age × Ln Total Cholesterol	5.0969	N/A
	Ln HDL–C (mg/dL)	14.2871	−0.4596
	Ln Age × Ln HDL–C	−3.6424	N/A
	Ln Treated SBP (mm Hg)	3.3128	3.3088
	Ln Untreated SBP (mm Hg)	3.2339	3.2282
	Current Smoker (1 = Yes, 0 = No)	−2.9244	−0.4882
	Ln Age × Current Smoker	0.6021	N/A
	Diabetes (1 = Yes, 0 = No)	0.6608	0.6518
	**Mean (Coefficient × Value)**	**−19.22938**	**32.45731**
	**Baseline Survival (S_5_)**	**0.9915944**	**0.9914698**

Abbreviations: SBP, systolic blood pressure; HDL-C, high-density lipoprotein cholesterol; Ln, natural logarithm; N/A, covariate was not included in the equation.

**Table 5:** Sensitivity, specificity and ability of discrimination and calibration for recalibrated the FRS, SCORE, CN-ICVD and PCE models for 5-year cardiovascular mortality.

Models	Cut-off^#^ (%)	Sensitivity (95%CI)	Specificity (95%CI)	+LR^*^	−LR^**^	AUC (95%CI)	χ^2^	*P*
**general-FRS**								
Men	1.16	80.00 (69.6–88.1)	62.23 (60.7, 63.8)	2.12	0.32	0.776 (0.763, 0.789)	4.032	0.402
Women	1.14	73.86 (63.4–82.7)	70.93 (69.8–72.0)	2.54	0.37	0.756 (0.746, 0.767)	9.448	0.051
**simplified-FRS**								
Men	1.17	82.50 (72.4, 90.1)	62.77 (61.2, 64.3)	2.22	0.28	0.795 (0.782, 0.807)	1.160	0.798
Women	0.73	87.50 (78.7, 93.6)	55.03 (53.8, 56.3)	1.95	0.23	0.753 (0.742, 0.763)	24.735	<0.001
**SCORE-low**								
Men	1.16	65.00 (53.5–75.3)	70.94 (69.5–72.4)	2.24	0.49	0.733 (0.719, 0.747)	22.430	<0.001
Women	0.2	79.07 (69.0, 87.1)	61.08 (59.8, 62.3)	2.03	0.34	0.743 (0.732, 0.754)	35.675	<0.001
**SCORE-high**								
Men	1.44	78.75 (68.2, 87.1)	57.21 (55.6–58.8)	1.84	0.37	0.731 (0.717, 0.745)	5.109	0.276
Women	0.27	79.55 (69.6–87.4)	60.41 (59.2–61.6)	2.01	0.34	0.739 (0.728, 0.749)	28.819	<0.001
**CN-ICVD**								
Men	1.03	82.50 (72.4, 90.1)	63.98 (62.4, 65.5)	2.29	0.27	0.793 (0.780, 0.805)	2.917	0.405
Women	1.01	73.86 (63.4, 82.7)	68.00 (66.8, 69.2)	2.31	0.38	0.755 (0.745, 0.766)	5.375	0.251
**PCE-white**								
Men	1.02	85.00 (75.3, 92.0)	57.90 (56.3, 59.5)	2.02	0.26	0.779 (0.766, 0.792)	12.513	0.014
Women	0.99	77.27 (67.1–85.5)	67.05 (65.9, 68.2)	2.35	0.34	0.758 (0.747, 0.768)	4.572	0.334
**PCE-AA**								
Men	1.16	80.00 (69.6, 88.1)	62.23 (60.7, 63.8)	2.12	0.32	0.776 (0.763, 0.789)	4.032	0.402
Women	1.02	73.86 (63.4, 82.7)	69.36 (68.2, 70.5)	2.41	0.38	0.760 (0.750, 0.771)	10.720	0.030

^#^The cut off recommended by present population. *+LR, Positive likelihood ratio. ^##^–LR, Negative likelihood ratio.

## References

[b1] Yusuf S. . Effect of potentially modifiable risk factors associated with myocardial infarction in 52 countries (the INTERHEART study): case-control study. Lancet 364, 937–952, doi: 10.1016/S0140-6736(04)17018-9 (2004). 15364185

[b2] Michos E. D. & Blumenthal R. S. How accurate are 3 risk prediction models in US women? Circulation 125, 1723–1726, doi: 10.1161/CIRCULATIONAHA.112.099929 (2012). 22399534

[b3] Lloyd-Jones D. M. Cardiovascular risk prediction: basic concepts, current status, and future directions. Circulation 121, 1768–1777, doi: 10.1161/CIRCULATIONAHA.109.849166 (2010). 20404268

[b4] D’Agostino R. B. Sr. . General cardiovascular risk profile for use in primary care: the Framingham Heart Study. Circulation 117, 743–753, doi: 10.1161/CIRCULATIONAHA.107.699579 (2008). 18212285

[b5] Conroy R. M. . Estimation of ten-year risk of fatal cardiovascular disease in Europe: the SCORE project. Eur Heart J 24, 987–1003, doi: 10.1016/s0195-668x(03)00114-3 (2003). 12788299

[b6] Goff D. C. Jr. . 2013 ACC/AHA guideline on the assessment of cardiovascular risk: a report of the American College of Cardiology/American Heart Association Task Force on Practice Guidelines. Circulation 129, S49–73, doi: 10.1161/01.cir.0000437741.48606.98 (2014). 24222018

[b7] Carroll S. J. . Validation of continuous clinical indices of cardiometabolic risk in a cohort of Australian adults. BMC Cardiovascular Disorders 14, 1–9, doi: 10.1186/1471-2261-14-27 (2014). 24571233 PMC3976083

[b8] Artigao-Rodenas L. M. . Framingham Risk Score for Prediction of Cardiovascular Diseases: A Population-Based Study from Southern Europe. PLoS ONE 8, e73529, doi: 10.1371/journal.pone.0073529 (2013). 24039972 PMC3764050

[b9] Bozorgmanesh M., Hadaegh F. & Azizi F. Predictive accuracy of the ‘Framingham’s general CVD algorithm’ in a Middle Eastern population: Tehran Lipid and Glucose Study. International journal of clinical practice 65, 264–273, doi: 10.1111/j.1742-1241.2010.02529.x (2011). 21314863

[b10] Chia Y. C., Gray S. Y., Ching S. M., Lim H. M. & Chinna K. Validation of the Framingham general cardiovascular risk score in a multiethnic Asian population: a retrospective cohort study. BMJ Open 5, e007324, doi: 10.1136/bmjopen-2014-007324 (2015). PMC4442208 25991451

[b11] Cook N. R. . Comparison of the Framingham and Reynolds Risk scores for global cardiovascular risk prediction in the multiethnic Women’s Health Initiative. Circulation 125, 1748–1756, s1741–1711, doi: 10.1161/circulationaha.111.075929 (2012). 22399535 PMC3324658

[b12] Selvarajah S. . Comparison of the Framingham Risk Score, SCORE and WHO/ISH cardiovascular risk prediction models in an Asian population. Int J Cardiol 176, 211–218, doi: 10.1016/j.ijcard.2014.07.066 (2014). 25070380

[b13] Jorstad H. T. . The Systematic COronary Risk Evaluation (SCORE) in a large UK population: 10-year follow-up in the EPIC-Norfolk prospective population study. Eur J Prev Cardiol 22, 119–126, doi: 10.1177/2047487313503609 (2015). 24002125

[b14] Aspelund T., Thorgeirsson G., Sigurdsson G. & Gudnason V. Estimation of 10-year risk of fatal cardiovascular disease and coronary heart disease in Iceland with results comparable with those of the Systematic Coronary Risk Evaluation project. Eur J Cardiovasc Prev Rehabil 14, 761–768, doi: 10.1097/HJR.0b013e32825fea6d (2007). 18043296

[b15] Lindman A. S. . The ability of the SCORE high-risk model to predict 10-year cardiovascular disease mortality in Norway. Eur J Cardiovasc Prev Rehabil 14, 501–507, doi: 10.1097/HJR.0b013e328011490a (2007). 17667638

[b16] Hu D. & Yu D. Epidemiology of cardiovascular disease in Asian women. Nutrition, Metabolism and Cardiovascular Diseases 20, 394–404, doi: 10.1016/j.numecd.2010.02.016 (2010). 20591635

[b17] Jung K. J. . The ACC/AHA 2013 pooled cohort equations compared to a Korean Risk Prediction Model for atherosclerotic cardiovascular disease. Atherosclerosis 242, 367–375, doi: 10.1016/j.atherosclerosis.2015.07.033 (2015). 26255683

[b18] Chia Y. C., Lim H. M. & Ching S. M. Validation of the pooled cohort risk score in an Asian population - a retrospective cohort study. BMC Cardiovasc Disord 14, 163, doi: 10.1186/1471-2261-14-163 (2014). 25410585 PMC4246627

[b19] Zhang M. . Development and Validation of a Risk-Score Model for Type 2 Diabetes: A Cohort Study of a Rural Adult Chinese Population. PLoS One 11, e0152054, doi: 10.1371/journal.pone.0152054 (2016). 27070555 PMC4829145

[b20] Perloff D. . Human blood pressure determination by sphygmomanometry. Circulation 88, 2460–2470 (1993). 8222141 10.1161/01.cir.88.5.2460

[b21] Hypertension W. G. o. C. G. f. t. M. o. 2010 Chinese Guidelines for the Management of Hypertension. Chinese Journal of Cardiology 39, 579–616, doi: 10.3760/cma.j.issn.0253-3758.2011.07.002 (2011). 22088239

[b22] American Diabetes A. Diagnosis and classification of diabetes mellitus. Diabetes Care 32 Suppl 1, S62–67, doi: 10.2337/dc09-S062 (2009). 19118289 PMC2613584

[b23] Stone N. J. . 2013 ACC/AHA guideline on the treatment of blood cholesterol to reduce atherosclerotic cardiovascular risk in adults: a report of the American College of Cardiology/American Heart Association Task Force on Practice Guidelines. J Am Coll Cardiol 63, 2889–2934, doi: 10.1016/j.jacc.2013.11.002 (2014). 24239923

[b24] Wu Y. . Estimation of 10-year risk of fatal and nonfatal ischemic cardiovascular diseases in Chinese adults. Circulation 114, 2217–2225, doi: 10.1161/CIRCULATIONAHA.105.607499 (2006). 17088464

[b25] Graham I. . European guidelines on cardiovascular disease prevention in clinical practice: executive summary. Atherosclerosis 194, 1–45, doi: 10.1016/j.atherosclerosis.2007.08.024 (2007). 17880983

[b26] D’Agostino R. B. & Nam B. H. Evaluation of the Performance of Survival Analysis Models: Discrimination and Calibration Measures. Handbook of Statistics 23, 1–25, doi: 10.1016/S0169-7161(03)23001-7 (2003).

[b27] Demler O. V., Paynter N. P. & Cook N. R. Tests of calibration and goodness-of-fit in the survival setting. Stat Med 34, 1659–1680, doi: 10.1002/sim.6428 (2015). 25684707 PMC4555993

[b28] Lemeshow S. & Hosmer D. W. Jr. A review of goodness of fit statistics for use in the development of logistic regression models. Am J Epidemiol 115, 92–106 (1982). 7055134 10.1093/oxfordjournals.aje.a113284

[b29] Muntner P. . Validation of the atherosclerotic cardiovascular disease Pooled Cohort risk equations. Jama 311, 1406–1415, doi: 10.1001/jama.2014.2630 (2014). 24682252 PMC4189930

[b30] Menotti A., Lanti M., Puddu P. E. & Kromhout D. Coronary heart disease incidence in northern and southern European populations: a reanalysis of the seven countries study for a European coronary risk chart. Heart 84, 238–244 (2000). 10956281 10.1136/heart.84.3.238 PMC1760967

[b31] Menotti A., Puddu P. E. & Lanti M. Comparison of the Framingham risk function-based coronary chart with risk function from an Italian population study. Eur Heart J 21, 365–370, doi: 10.1053/euhj.1999.1864 (2000). 10666350

