# Supplementary material for: Comparison of validation and application on various cardiovascular disease mortality risk prediction models in Chinese rural population
Source: Sci Rep. 2017 Mar 24;7:43227. doi: 10.1038/srep43227 (PMC5364500; doi:10.1038/srep43227)
Supplement: Supplementary Information [file srep43227-s1.pdf]

Title:

Comparison of validation and application on various cardiovascular disease mortality risk prediction models in Chinese rural population

Authors:

Changqing Sun[1*], Fei Xu[1], Xiaotian Liu[2], Mingwang Fang[3], Hao Zhou[2], Yixiao Lian[1], Chen Xie[1], Nan Sun[4] and Chongjian Wang [2*]

Authors affiliations:

[1] Department of Social Medicine and Health Management, College of Public Health, Zhengzhou University, Zhengzhou, Henan, *PR* China.

[2] Department of Epidemiology and Biostatistics, College of Public Health, Zhengzhou University, Zhengzhou, Henan, *PR* China.

[3]Department of Health Education, West China School of Public Health, Sichuan University, Chengdu, Sichuan, *PR* China.

[4]Department of Management Information Systems, Terry College of Business, University of Georgia, Athens, Georgia, United State.

*** Corresponding author**

Prof. Chang-qing Sun & Dr. Chongjian Wang

Department of Social Medicine and Health Management,

College of Public Health, Zhengzhou University

100 Kexue Avenue, Zhengzhou, 450001, Henan, *PR* China

Phone: +86 371 67781926;

Fax: +86 371 67781919

E-mail: suncq@zzu.edu.cn & tjwcj2005@126.com

Supplementary Table S1 The cardiovascular risk categorization of PEC models compared with that of FRS, SCORE and CN-ICVD models.

Models	PCE-white model					PCE-AA model				
	<7.5%	7.5-9.9%	10.0-19.9%	≥20%	Total	<7.5%	7.5-9.9%	10.0-19.9%	≥20%	Total
**General-FRS**										
<10%	6280 (97.03)	493 (45.82)	89 (4.32)	0 (0.00)	**6862**	5215 (99.90)	1006 (78.23)	632 (24.62)	9 (7.12)	**6862**
10-20%	192 (2.97)	582 (54.09)	1593 (77.25)	13 (1.79)	**2380**	5 (0.10)	280 (21.77)	1688 (65.76)	407 (32.17)	**2380**
>20%	0 (0.00)	1 (0.09)	380 (18.43)	715 (98.21)	**1096**	0 (0.00)	0 (0.00)	247 (9.62)	849 (67.11)	**1096**
**Total**	**6472**	**1076**	**2062**	**728**	**10338**	**5220**	**1286**	**2567**	**1265**	**10338**
**Simplified-FRS**										
<10%	6089 (94.08)	483 (44.89)	143 ((6.93)	1 (0.14)	**6716**	5216 (99.92)	935 (72.71)	553 (21.54)	12 (0.95)	**6716**
10-20%	382 (5.90)	577 (53.62)	1464 (71.00)	56 (7.69)	**2479**	4 (0.08)	351 (27.29)	1572 (68.25)	372 (29.41)	**2479**
>20%	1 (0.02)	16 (1.49)	455 (22.07)	671 (92.17)	**1143**	0 (0.00)	0 (0.00)	262 (10.21)	881 (69.64)	**1143**
**Total**	**6472**	**1076**	**2062**	**728**	**10338**	**5220**	**1286**	**2567**	**1265**	**10338**
**SCORE-low**										
<1%	5822(89.96)	198 (18.40)	39 (1.89)	0 (0.00)	**6059**	5011 (96.00)	518 (40.28)	447 (7.60)	83 (6.56)	**6059**
1-5%	650(10.04)	878 (81.60)	1829 (88.40)	140 (19.23)	**3497**	209 (4.00)	768 (59.72)	1925 (74.99)	595 (47.04)	**3497**
≥5%	0 (0.00)	0 (0.00)	194 (9.41)	588 (80.77)	**782**	0 (0.00)	0 (0.00)	195 (17.41)	587 (46.40)	**782**
**Total**	**6472**	**1076**	**2062**	**728**	**10338**	**5220**	**1286**	**2567**	**1265**	**10338**
**SCORE-high**										
<1%	4709 (72.76)	29 (2.70)	5 (0.24)	0 (0.00)	**4743**	4269 (81.78)	210 (16.33)	217 (8.45)	47 (3.72)	**4743**
1-5%	1761 (27.21)	1031 (94.14)	1117 (54.17)	17 (2.34)	**3908**	951 (18.22)	1070 (83.20)	1511 (58.86)	376 (29.72)	**3907**
≥5%	2 (0.03)	34 (3.16)	940 (45.59)	711 (97.66)	**1687**	0 (0.00)	6 (0.47)	839 (32.69)	842 (66.56)	**1687**
**Total**	**6472**	**1076**	**2062**	**728**	**10338**	**5220**	**1286**	**2657**	**1265**	**10338**
**CN-ICVD**										
<5%	6463 (99.86)	1058 (98.33)	1789 (86.76)	199 (27.33)	**9509**	5220 (100.00)	1283 (99.77)	2399 (93.46)	607 (47.98)	**9509**
5-10%	8 (0.12)	18 (1.67)	254 (12.32)	338 (46.43)	**618**	0 (0.00)	3 (0.23)	159 (6.19)	456 (36.05)	**618**
≥10%	1 (0.02)	0 (0.00)	19 (0.92)	191 (26.34)	**211**	0 (0.00)	0 (0.00)	9 (0.35)	202 (15.97)	**211**
**Total**	**6472**	**1076**	**2062**	**728**	**10338**	**5220**	**1286**	**2567**	**1265**	**10338**

Supplementary Table S2 The cardiovascular risk categorization of PEC models compared with that of FRS, SCORE and CN-ICVD models.

Models	PCE-white model					PCE-AA model				
	<7.5%	7.5-9.9%	10.0-19.9%	≥20%	Total	<7.5%	7.5-9.9%	10.0-19.9%	≥20%	Total
**General-FRS**										
<10%	6280 (97.03)	493 (45.82)	89 (4.32)	0 (0.00)	**6862**	5215 (99.90)	1006 (78.23)	632 (24.62)	9 (7.12)	**6862**
10-20%	192 (2.97)	582 (54.09)	1593 (77.25)	13 (1.79)	**2380**	5 (0.10)	280 (21.77)	1688 (65.76)	407 (32.17)	**2380**
>20%	0 (0.00)	1 (0.09)	380 (18.43)	715 (98.21)	**1096**	0 (0.00)	0 (0.00)	247 (9.62)	849 (67.11)	**1096**
**Total**	**6472**	**1076**	**2062**	**728**	**10338**	**5220**	**1286**	**2567**	**1265**	**10338**
**Simplified-FRS**										
<10%	6089 (94.08)	483 (44.89)	143 ((6.93)	1 (0.14)	**6716**	5216 (99.92)	935 (72.71)	553 (21.54)	12 (0.95)	**6716**
10-20%	382 (5.90)	577 (53.62)	1464 (71.00)	56 (7.69)	**2479**	4 (0.08)	351 (27.29)	1572 (68.25)	372 (29.41)	**2479**
>20%	1 (0.02)	16 (1.49)	455 (22.07)	671 (92.17)	**1143**	0 (0.00)	0 (0.00)	262 (10.21)	881 (69.64)	**1143**
**Total**	**6472**	**1076**	**2062**	**728**	**10338**	**5220**	**1286**	**2567**	**1265**	**10338**
**SCORE-low**										
<1%	5822(89.96)	198 (18.40)	39 (1.89)	0 (0.00)	**6059**	5011 (96.00)	518 (40.28)	447 (7.60)	83 (6.56)	**6059**
1-5%	650(10.04)	878 (81.60)	1829 (88.40)	140 (19.23)	**3497**	209 (4.00)	768 (59.72)	1925 (74.99)	595 (47.04)	**3497**
≥5%	0 (0.00)	0 (0.00)	194 (9.41)	588 (80.77)	**782**	0 (0.00)	0 (0.00)	195 (17.41)	587 (46.40)	**782**
**Total**	**6472**	**1076**	**2062**	**728**	**10338**	**5220**	**1286**	**2567**	**1265**	**10338**
**SCORE-high**										
<1%	4709 (72.76)	29 (2.70)	5 (0.24)	0 (0.00)	**4743**	4269 (81.78)	210 (16.33)	217 (8.45)	47 (3.72)	**4743**
1-5%	1761 (27.21)	1031 (94.14)	1117 (54.17)	17 (2.34)	**3908**	951 (18.22)	1070 (83.20)	1511 (58.86)	376 (29.72)	**3907**
≥5%	2 (0.03)	34 (3.16)	940 (45.59)	711 (97.66)	**1687**	0 (0.00)	6 (0.47)	839 (32.69)	842 (66.56)	**1687**
**Total**	**6472**	**1076**	**2062**	**728**	**10338**	**5220**	**1286**	**2657**	**1265**	**10338**
**CN-ICVD**										
<5%	6463 (99.86)	1058 (98.33)	1789 (86.76)	199 (27.33)	**9509**	5220 (100.00)	1283 (99.77)	2399 (93.46)	607 (47.98)	**9509**
5-10%	8 (0.12)	18 (1.67)	254 (12.32)	338 (46.43)	**618**	0 (0.00)	3 (0.23)	159 (6.19)	456 (36.05)	**618**
≥10%	1 (0.02)	0 (0.00)	19 (0.92)	191 (26.34)	**211**	0 (0.00)	0 (0.00)	9 (0.35)	202 (15.97)	**211**
**Total**	**6472**	**1076**	**2062**	**728**	**10338**	**5220**	**1286**	**2567**	**1265**	**10338**

Supplementary Table S3 The cardiovascular risk categorization of PEC models compared with that of FRS, SCORE and CN-ICVD models in men.

Models	PCE-white model					PCE-AA model				
	**<7.5%**	**7.5-9.9%**	**10.0-19.9%**	**≥20%**	**Total**	**<7.5%**	**7.5-9.9%**	**10.0-19.9%**	**≥20%**	**Total**
**General-FRS**										
<10%	1522 (98.58)	248 (43.89)	13 (1.00)	0 (0.00)	**1783**	1247 (99.60)	477 (63.43)	59 (4.25)	0 (0.00)	**1783**
10-20%	22 (1.42)	317 (56.11)	1052 (81.17)	4 (0.74)	**1395**	5 (0.40)	275 (36.57)	1082 (77.95)	33 (5.97)	**1395**
>20%	0 (0.00)	0 (0.00)	231 (17.83)	536 (99.26)	**767**	0 (0.00)	0 (0.00)	247 (17.80)	520 (94.03)	**767**
**Total**	**1544**	**565**	**1296**	**540**	**3945**	**1252**	**752**	**1388**	**553**	**3945**
**Simplified-FRS**										
<10%	1409 (91.26)	231 (40.89)	55 (4.25)	1 (0.19)	**1696**	1250 (99.84)	427 (56.78)	19 (1.37)	0 (0.00)	**1696**
10-20%	135 (8.74)	332 (58.76)	946 (72.99)	37 (6.85)	**1450**	2 (0.16)	325 (43.22)	1109 (79.90)	14 (2.53)	**1450**
>20%	0 (0.00)	2 (0.35)	295 (22.76)	502 (92.96)	**799**	0 (0.00)	0 (0.00)	260 (18.73)	539 (97.47)	**799**
**Total**	**1544**	**565**	**1296**	**540**	**3945**	**1252**	**752**	**1388**	**553**	**3945**
**SCORE-low**										
<1%	1161(75.19)	71 (12.57)	13 (1.00)	0 (0.00)	**1245**	1075 (85.86)	144 (19.15)	26 (1.87)	0 (0.00)	**1245**
1-5%	383(24.81)	494 (87.43)	1164 (89.81)	494 (83.52)	**2130**	177 (14.14)	608 (80.85)	1173 (84.51)	172(31.10)	**2130**
≥5%	0 (0.00)	0 (0.00)	119 (9.18)	71 (16.48)	**570**	0 (0.00)	0 (0.00)	189 (13.62)	381(68.90)	**570**
**Total**	**1544**	**565**	**1296**	**540**	**3945**	**1252**	**752**	**1388**	**553**	**3945**
**SCORE-high**										
<1%	562 (36.40)	2 (0.35)	0 (0.00)	0 (0.00)	**564**	553 (44.17)	8 (1.06)	3 (0.22)	0 (0.00)	**564**
1-5%	981 (63.54)	552 (97.70)	535 (41.28)	3 (0.56)	**2071**	699 (55.83)	738 (98.14)	602 (43.37)	32 (5.79)	**2071**
≥5%	1 (0.06)	11 (1.95)	761 (58.72)	537 (99.44)	**1310**	0 (0.00)	6 (0.80)	783 (56.41)	521 (94.21)	**1310**
**Total**	**1544**	**565**	**1296**	**540**	**3945**	**1252**	**752**	**1388**	**553**	**3945**
**CN-ICVD**										
<5%	1536 (99.48)	559 (98.94)	1171 (90.35)	168 (31.11)	**3434**	1252 (100.00)	749 (99.60)	1234 (88.90)	199 (35.99)	**3434**
5-10%	7 (0.45)	6 (1.06)	113 (8.72)	243 (45.00)	**369**	0 (0.00)	3 (0.40)	145 (10.45)	221 (39.96)	**369**
≥10%	1 (0.07)	0 (0.00)	12 (0.93)	129 (23.89)	**142**	0 (0.00)	0 (0.00)	9 (0.65)	199 (24.05)	**142**
**Total**	**1544**	**565**	**1296**	**540**	**3945**	**1252**	**752**	**1388**	**553**	**3945**

Supplementary Table S4 Agreement of cardiovascular risk categorization and correlation of scores for the FRS, SCORE and CN-ICVD prediction models.

**Models**	Both high or both low (n)	Both high (n)	First model* high/ comparator low (n)	First model* high/ comparator low (n)	**ρ***
**general-FRS* and simplified-FRS**					
Overall	7354	921	0	1	0.874
Men	2211	652	0	0	0.862
Women	5143	269	0	1	0.851
**general-FRS* and SCORE-low**					
Overall	6426	653	2	3	0.766
Men	1696	496	0	0	0.767
Women	4730	157	2	3	0.695
**general-FRS* and SCORE-high**					
Overall	5603	954	0	23	0.733
Men	1294	731	0	5	0.760
Women	4309	223	0	18	0.665
**general-FRS* and CN-ICVD**					
Overall	7065	205	430	0	0.507
Men	1919	138	329	0	0.524
Women	5146	67	101	0	0.502
**simplified-FRS* and SCORE-low**					
Overall	6435	674	0	0	0.786
Men	1726	519	0	0	0.795
Women	4709	155	0	0	0.707
**simplified-FRS* and SCORE-high**					
Overall	5622	981	0	11	0.744
Men	1317	754	0	2	0.758
Women	4305	227	0	9	0.679
**simplified-FRS* and CN-ICVD**					
Overall	6922	207	442	0	0.506
Men	1833	138	343	0	0.529
Women	5089	69	99	0	0.497
**SCORE-low* and SCORE-high**					
Overall	5525	782	0	0	0.827
Men	1134	570	0	0	0.735
Women	4391	212	0	0	0.812
**SCORE-low* and CN-ICVD**					
Overall	5253	194	248	0	0.457
Men	1377	132	195	0	0.503
Women	4876	62	53	0	0.446
**SCORE-high* and CN-ICVD**					
Overall	4953	210	981	0	0.421
Men	705	141	825	0	0.462
Women	4248	69	156	0	0.397

***ρ, Spearman's correlation coefficient**

Supplementary Table S5 Calibration of Kaplan-Meier Observed and Predicted 5-year Cardiovascular Disease (CVD) Death Event in Men Using SCORE models (SCORE-low and SCORE-high) and PCE models (PCE-white and PCE-AA).

**Groups**	**Actual events***	**Kaplan-Meier adjusted events†**	**Predicted events‡**	**Calibration χ^2**	***P* value**
**SCORE-low model**					
1	2	2.229	1.081		
2	7	12.202	2.696		
3	12	16.227	5.090	22.430	<0.001
4	20	21.517	9.143		
5	39	52.262	24.443		
**SCORE-high model**					
1	2	2.231	2.177		
2	7	12.020	5.260		
3	12	16.360	9.685	5.109	0.276
4	22	23.898	17.096		
5	37	49.789	44.818		
**PCE-white model**					
1	6	9.609	9.571		
2	5	8.548	20.728		
3	10	10.813	32.822	94.735	<0.001
4	22	23.676	49.370		
5	37	49.577	91.896		
**PCE-AA model**					
1	4	4.480	2.970		
2	9	16.266	5.144		
3	5	5.385	8.036	14.868	0.005
4	21	25.389	12.041		
5	41	50.617	30.248		

* **Actual number of events through follow-up period;**

† **Observed number of events after Kaplan-Meier adjustment through follow-up period;**

‡ **Predicted number of events based on the different models through follow-up period.**

Supplementary Table S6 Calibration of Kaplan-Meier Observed and Predicted 5-year Cardiovascular Disease (CVD) Death Event in Women Using SCORE models (SCORE-low and SCORE-high) and PCE models (PCE-white and PCE-AA).

**Groups**	**Actual events***	**Kaplan-Meier adjusted events†**	**Predicted events‡**	**Calibration χ^2**	***P* value**
**SCORE-low model**					
1	5	24.741	0.174		
2	4	7.425	0.551		
3	10	13.812	1.511	35.675	<0.001
4	21	26.169	3.615		
5	48	70.745	16.436		
**SCORE-high model**					
1	5	24.725	0.287		
2	4	7.427	0.884		
3	9	12.779	2.363	28.819	<0.001
4	23	28.168	5.539		
5	47	69.892	24.772		
**PCE-white model**					
1	4	21.400	3.667		
2	7	14.040	7.823		
3	11	18.100	14.062	2.310	0.679
4	19	24.150	25.718		
5	47	65.040	63.916		
**PCE-AA model**					
1	4	21.680	3.300		
2	7	14.310	5.142		
3	8	11.400	8.292	12.159	0.016
4	25	30.990	15.349		
5	44	64.940	38.145		

* **Actual number of events through follow-up period;**

† **Observed number of events after Kaplan-Meier adjustment through follow-up period;**

‡ **Predicted number of events based on the different models through follow-up period.**

Supplementary Table S7 Calibration of Kaplan-Meier Observed and Predicted 5-year Cardiovascular Disease (CVD) Death Event in Men Using the Recalibrated FRS models (general-FRS and simplified-FRS), CN-ICVD and PCE models (PCE-white and PCE-AA).

**Groups**	**Actual events***	**Kaplan-Meier adjusted events†**	**Predicted events‡**	**Calibration χ^2**	***P* value**
**general-FRS model**					
1	2	2.264	3.222		
2	3	5.104	6.717		
3	11	15.369	12.338	4.032	0.402
4	19	30.525	22.132		
5	45	51.372	62.623		
**simplified-FRS model**					
1	2	2.251	3.276		
2	2	3.909	6.831		
3	10	14.640	12.087	1.660	0.798
4	17	23.979	21.816		
5	49	59.556	61.362		
**CN-ICVD model**					
1	2	2.256	4.551		
2	8	13.813	9.610		
3	15	16.449	19.072	2.917	0.405
4	55	72.519	72.896		
5	-	-	-		
**PCE-white model**					
1	2	2.290	3.110		
2	2	2.247	6.711		
3	13	19.083	12.235	12.513	0.014
4	18	29.726	21.961		
5	45	51.252	63.079		
**PCE-AA model**					
1	2	2.264	3.222		
2	3	5.104	6.717		
3	11	15.369	12.338	4.032	0.402
4	19	30.525	22.132		
5	45	51.372	62.623		

* **Actual number of events through follow-up period;**

† **Observed number of events after Kaplan-Meier adjustment through follow-up period;**

‡ **Predicted number of events based on the different models through follow-up period.**

Supplementary Table S8 Calibration of Kaplan-Meier Observed and Predicted 5-year Cardiovascular Disease (CVD) Death Event in Women Using the Recalibrated FRS models (general-FRS and simplified-FRS), CN-ICVD and PCE models (PCE-white and PCE-AA).

**Groups**	**Actual events***	**Kaplan-Meier adjusted events†**	**Predicted events‡**	**Calibration χ^2**	***P* value**
**general-FRS model**					
1	4	23.020	5.041		
2	6	13.270	10.224		
3	10	10.040	17.863	9.448	0.051
4	21	29.870	32.172		
5	47	66.940	85.627		
**simplified-FRS model**					
1	5	23.208	5.334		
2	5	11.638	10.645		
3	7	7.084	18.575	24.735	<0.001
4	27	40.405	33.470		
5	44	59.883	85.231		
**CN-ICVD model**					
1	3	6.999	6.147		
2	7	28.253	11.383		
3	10	13.284	18.092	5.375	0.251
4	17	21.182	31.519		
5	51	71.877	84.670		
**PCE-white model**					
1	3	3.397	5.244		
2	7	30.403	10.286		
3	10	13.535	17.559	4.572	0.334
4	21	29.575	31.546		
5	47	67.385	86.129		
**PCE-AA model**					
1	4	22.434	6.311		
2	5	11.939	10.515		
3	9	9.053	17.236	10.720	0.030
4	23	32.432	30.272		
5	47	66.625	86.465		

* **Actual number of events through follow-up period;**

† **Observed number of events after Kaplan-Meier adjustment through follow-up period;**

‡ **Predicted number of events based on the different models through follow-up period.**

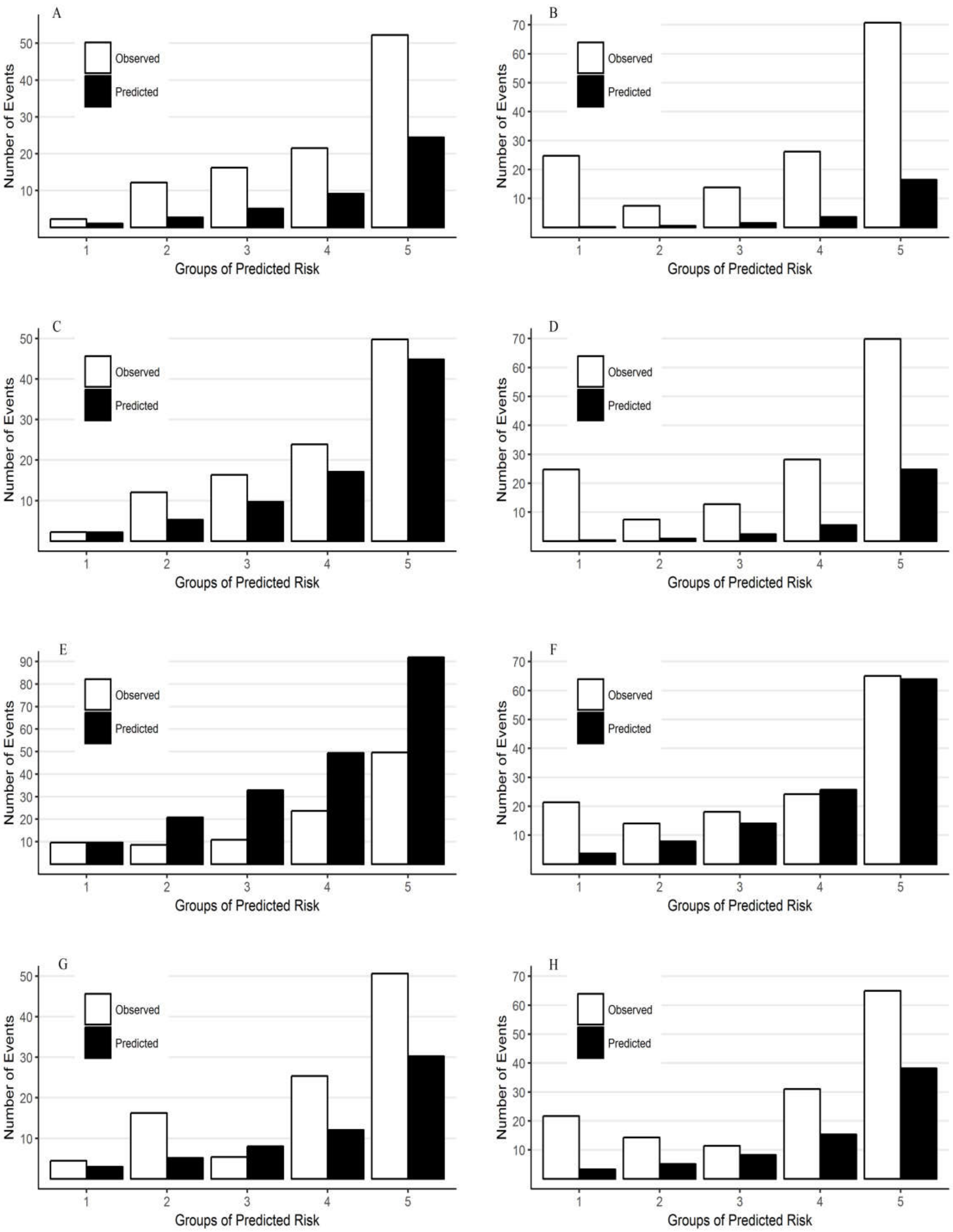

Supplementary Figure S1 Kaplan-Meier observed and predicted cardiovascular mortality events for SCORE-low (A for men, B for women), SCORE-high (C for men, D for women), PCE-white (E for men, F for women) and PCE-AA (G for men, H for women) cardiovascular risk prediction models, by groups of predicted probabilities.

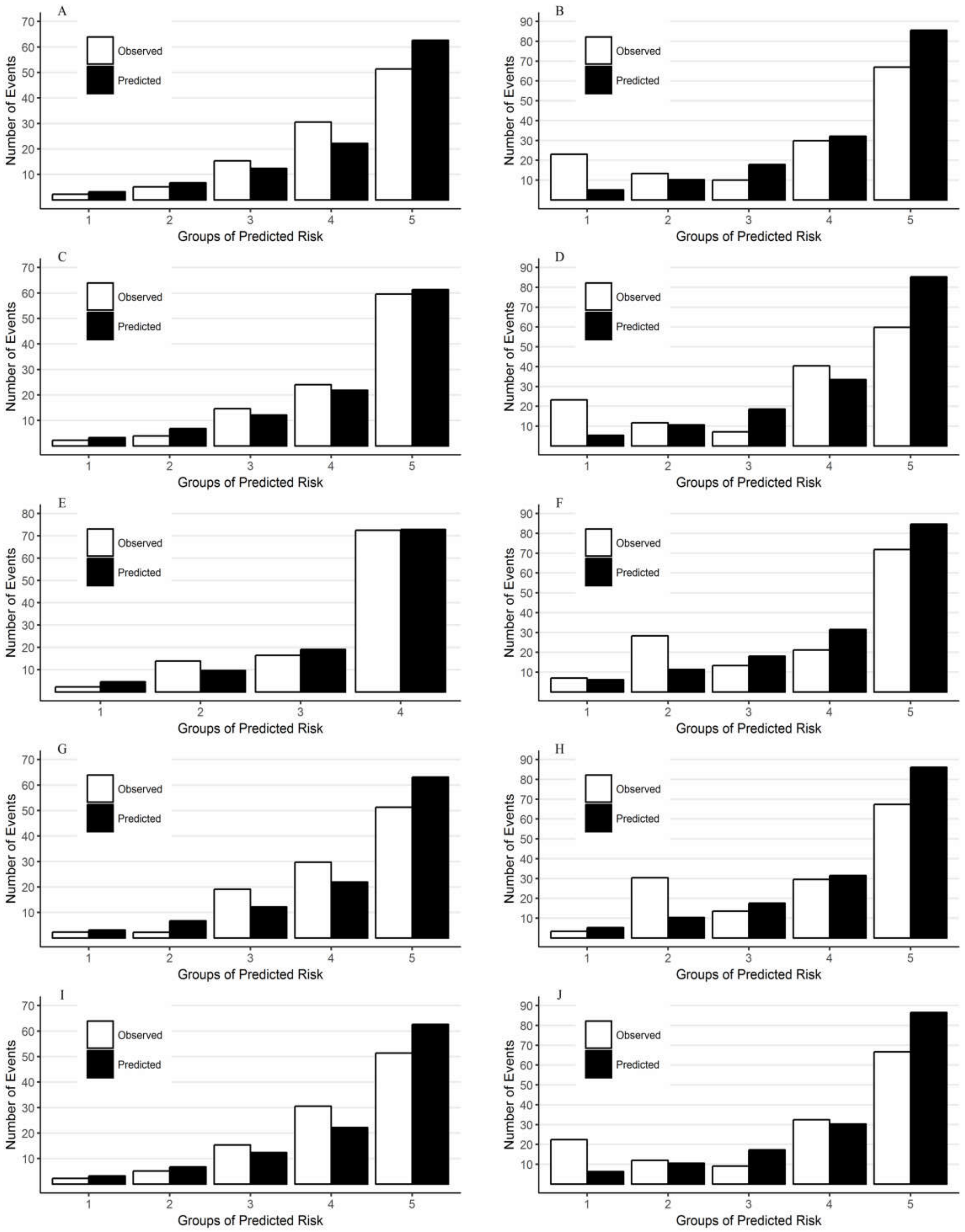

Supplementary Figure S2 Kaplan-Meier observed and predicted cardiovascular mortality events for recalibrated 5 models: General-FRS (A for men, B for women), simplified-FRS (C for men, D for women), CN-ICVD (E for men, F for women), PCE-white (G for men, H for women), and PCE-AA (I for men, J for women), by groups of predicted probabilities.
